# BipA Is Associated with Preventing Autoagglutination and Promoting Biofilm Formation in *Bordetella holmesii*

**DOI:** 10.1371/journal.pone.0159999

**Published:** 2016-07-22

**Authors:** Yukihiro Hiramatsu, Momoko Saito, Nao Otsuka, Eri Suzuki, Mineo Watanabe, Keigo Shibayama, Kazunari Kamachi

**Affiliations:** 1 Department of Bacteriology II, National Institute of Infectious Diseases, Tokyo, Japan; 2 Graduate School of Infection Control Sciences, Kitasato University, Tokyo, Japan; Instituto Butantan, BRAZIL

## Abstract

*Bordetella holmesii* causes both invasive and respiratory diseases in humans. Although the number of cases of pertussis-like respiratory illnesses due to *B*. *holmesii* infection has increased in the last decade worldwide, little is known about the virulence factors of the organism. Here, we analyzed a *B*. *holmesii* isolate that forms large aggregates and precipitates in suspension, and subsequently demonstrated that the autoagglutinating isolate is deficient in Bordetella intermediate protein A (BipA) and that this deletion is caused by a frame-shift mutation in the *bipA* gene. A BipA-deficient mutant generated by homologous recombination also exhibited the autoagglutination phenotype. Moreover, the BipA mutant adhered poorly to an abiotic surface and failed to form biofilms, as did two other *B*. *holmesii* autoagglutinating strains, ATCC 51541 and ATCC 700053, which exhibit transcriptional down-regulation of *bipA* gene expression, indicating that autoagglutination indirectly inhibits biofilm formation. In a mouse intranasal infection model, the BipA mutant showed significantly lower levels of initial lung colonization than did the parental strain (*P* < 0.01), suggesting that BipA might be a critical virulence factor in *B*. *holmesii* respiratory infection. Together, our findings suggest that BipA production plays an essential role in preventing autoagglutination and indirectly promoting biofilm formation by *B*. *holmesii*.

## Introduction

*Bordetella holmesii* is a Gram-negative coccobacillus that was first reported in 1995 [1]. The organism is associated with a variety of invasive infections, including bacteremia, septicemia, endocarditis, pneumonia, and septic arthritis, in patients with underlying medical conditions, such as asplenia and sickle cell anemia, and has been isolated from such patients’ blood, pericardial effusion, and synovial fluids [1–5]. *B*. *holmesii* has been detected in respiratory specimens, such as nasopharynx and sputum samples from patients with pertussis-like symptoms [6, 7], and therefore may also be responsible for causing a disease similar to pertussis (whooping cough) in otherwise healthy individuals. Indeed, *B*. *holmesii* has been isolated in patients with suspected pertussis in various regions worldwide, including Asia, North and South America, and Europe, and the number of reported cases of respiratory infection due to *B*. *holmesii* has increased over the last decade [8–13]. Notably, during pertussis outbreaks in Japan and the US, *B*. *holmesii* was found to co-circulate with *Bordetella pertussis*, the primary etiologic agent of pertussis [9, 13], indicating that *B*. *holmesii* can spread concurrently with *B*. *pertussis* epidemics. However, it is difficult to discriminate between *B*. *pertussis* and *B*. *holmesii* with routine PCR used for *B*. *pertussis* diagnosis, since the target sequence (insertion sequence IS*481*) is also present in the *B*. *holmesii* genome.

A previous study demonstrated that both whole-cell and acellular pertussis vaccines (ACVs) confer little protection against *B*. *holmesii* in a mouse intranasal infection model [14]. In agreement, data from an epidemiologic study of a pertussis outbreak resulting from both *B*. *pertussis* and *B*. *holmesii* infections showed that ACVs were less effective against *B*. *holmesii* than *B*. *pertussis* in humans [13]. Pertussis toxin (PT), filamentous hemagglutinin (FHA), and pertactin (PRN) derived from *B*. *pertussis* are used in ACVs as major antigens. In addition, certain ACVs also contain fimbriae (FIM) [15]. However, none of these proteins were detected in *B*. *holmesii* using specific antibodies raised against *B*. *pertussis* virulence factors [4]. Recently, genome analyses revealed that while *B*. *holmesii* clinical strains harbor a gene encoding an FHA-like protein, they lack genes encoding PT, PRN, FIM2, and FIM3 [16–18]. These findings imply that *B*. *holmesii* produces an FHA-like protein that is immunologically distinct from that of *B*. *pertussis*. In *B*. *pertussis*, FHA appears to be involved in immune-suppression and adherence of the bacterium to human ciliated respiratory epithelia [19, 20]; however, the role of FHA in the pathogenesis of *B*. *holmesii* is unclear. Indeed, despite the increasing number of *B*. *holmesii* infections associated with pertussis-like illness, little is known regarding the virulence factors of the organism.

In the present study, we characterized a *B*. *holmesii* isolate that forms large aggregates and precipitates in bacterial suspension (i.e., bacterial autoagglutination). Moreover, the autoagglutinating isolate failed to form biofilms. By studying 1D migration of the bacterial proteins and identifying differential bands, we demonstrated that a single protein, BipA, was expressed at undetectable levels in the autoagglutinating strain than in other *B*. *holmesii* isolates. BipA is a 150-kDa outer membrane ligand binding protein (alias Bordetella intermediate protein A), which was previously identified in the classical *Bordetella* species, *Bordetella bronchiseptica*, *Bordetella parapertussis*, and *B*. *pertussis* [21, 22]; however, this protein had yet to be identified and characterized in *B*. *holmesii*. We therefore generated a BipA-deficient mutant to investigate the relationship between BipA production and bacterial autoagglutination in *B*. *holmesii*. Furthermore, we attempted to gain insights into the potential role of BipA in *B*. *holmesii* virulence using an *in vitro* biofilm formation assay and a mouse intranasal infection model.

## Materials and Methods

### Bacterial strains and culture conditions

The *B*. *holmesii* strains used in this study are listed in Table 1. Strains BH1–BH5 were isolated from distinct individuals infected with *B*. *holmesii* in same area and mostly the same period during a pertussis outbreak [9]. In contrast, there were no epidemiological links between the other *B*. *holmesii* strains used. All strains were cultured on Bordet-Gengou (BG) agar plates (Difco, Franklin Lakes, NJ, USA) supplemented with 15% defibrinated horse blood at 36°C. Streptomycin (Sm) was added to BG agar plates (30 μg/ml) when necessary.

**Table 1:** Characteristics of *Bordetella holmesii* clinical and ATCC strains used in this study.

Strain	Isolation year	Origin	Country	Bacterial autoagglutination	BipA production	Biofilm formation	Mechanism of loss of BipA production	Reference
BH1	2011	Respiratory	Japan	−	+	+		[9]
BH2	2011	Respiratory	Japan	−	+	+		[9]
BH3	2011	Respiratory	Japan	−	+	+		[9]
BH4	2011	Respiratory	Japan	−	+	+		[9]
BH5	2011	Respiratory	Japan	−	+	+		[9]
BH6	2011	Respiratory	Japan	−	+	+		[23]
BH7	2010	Pericardial effusion	Japan	+	−	−	frame-shift mutation (c.2039delG)	[24]
BH8	2012	Synovial fluid	Japan	−	+	+		This study
BH9	2015	Blood	Japan	−	+	+		This study
ATCC 51541	1983	Blood	US	+	−	−	Transcriptional down-regulation	[1]
ATCC 700053	Unknown	Blood	Saudi Arabia	+	−	−	Transcriptional down-regulation	[1]

### Autoagglutination assay

*B*. *holmesii* strains were cultured on BG agar plates and then suspended in casamino acid solution (1% casamino acid, 0.6% NaCl [pH 7.1]) to an optical density at 650 nm (OD_650_) of 1.0. Bacterial suspensions were incubated for 4 h at 36°C in disposable cuvettes under static conditions, and the OD_650_ of each suspension was measured every 30 min. In addition, the cell morphology of *B*. *holmesii* was visualized after 4 h incubation using an Olympus IX83 microscope (Olympus, Tokyo, Japan).

### Biofilm assay

Biofilm formation was assessed by scanning-electron microscopy (SEM) as described previously [25], with minor modifications. Briefly, *B*. *holmesii* strains were cultured on BG agar plates and then suspended in modified Stainer-Scholte (mSS) broth [26] to an OD_650_ of 0.2. Bacterial suspensions (2 ml) were then cultured with shaking at 36°C for 24 h and diluted to an OD_650_ of 0.2 in mSS broth. The resulting suspensions (2 ml) were incubated at 36°C under static conditions on vertically submerged glass slides (6 × 8 mm) in 6-well plates. After culturing for 5 or 72 h, the bacteria were washed 3 times with phosphate-buffered saline (PBS), fixed with 2.5% glutaraldehyde and 2.5% paraformaldehyde, and then post-fixed with 1% osmium tetroxide. The fixed cells were dehydrated through a graded series of ethanol and freeze-dried. Subsequently, the adherence of cells to glass slides and biofilm growth at the air-liquid interface were visualized using a SEM SU6600 (Hitachi, Tokyo, Japan). In addition, the levels of biofilm formation were assessed by crystal violet assay analysis [27]. A 1 ml suspension of each *B*. *holmesii* strain (OD_650_ = 0.2) was incubated at 36°C under static conditions in a 5 ml polystyrene tube. After culturing for 24, 48, and 72 h, the biofilms were stained by treatment with 1.5 ml of a 0.1% crystal violet solution at room temperature for 30 min, and washed with distilled water. The remaining crystal violet was solubilized with 1.5 ml of 95% ethanol and quantified by measuring the OD_540_ using a Multiskan FC Microplate Reader (Thermo Fisher Scientific, Inc., Waltham, MA, USA).

### Production of antibodies specific to recombinant BipA (rBipA)

For production of antibodies specific to the N-terminal (R1, amino acids 42–556) and C-terminal (R3, amino acids 1275–1466) regions of *B*. *holmesii* BipA (GenBank: EMD74886.1), the BipA-R1 and -R3 regions were PCR amplified from the genomic DNA of *B*. *holmesii* strain BH2 using the bipA R1-F and bipA R1-R, and bipA R3-F and bipA R3-R primers (S1 Table), respectively, and cloned into the Cold Shock Expression Vector, pCold II DNA (TaKaRa Bio, Inc., Shiga, Japan), which was linearized with KpnI, using an In-fusion Advantage PCR Cloning Kit (Clontech Laboratories, Inc., Mountain View, CA, USA). The resulting vector constructs were transformed into *Escherichia coli* BL21 cells, and the expression of His-tagged rBipA-R1 and rBipA-R3 was subsequently induced by incubating with 0.5 mM isopropyl-β-D-thiogalactopyranoside (IPTG) at 15°C for 24 h. Proteins were then purified using a HisTrap FF Crude Kit (GE Healthcare UK Ltd., Little Chalfont, UK) and immunized with BALB/c mice (Japan SLC, Inc., Hamamatsu, Japan), respectively. The resulting anti-rBipA-R1 and anti-rBipA-R3 antisera were mixed and used for immunoblotting analyses. These experiments were approved by the Animal Research Committee of Kitasato University.

### Immunoblotting

*B*. *holmesii* strains were subcultured on BG agar. Total protein was extracted from bacterial cells with SDS-lysis buffer (62.5 mM Tris-HCl, 1% SDS, 10% glycerol, 5% 2-mercaptoethanol [pH 6.8]). Protein samples (2 μg) were separated by sodium dodecyl sulfate-polyacrylamide gel electrophoresis (SDS-PAGE; 10 to 20% gradient gels) and transferred to nitrocellulose membranes (Bio-Rad, Hercules, CA, USA). Membranes were incubated with the anti-BipA antisera followed by a horseradish peroxidase (HRP)-conjugated secondary antibody (Bio-Rad), and antigen-antibody complexes were visualized by enhanced chemiluminescence using a Western Lighting ECL Pro (PerkinElmer, Waltham, MA, USA) and an LAS-3000 Luminescent Image Analyzer (Fujifilm, Tokyo, Japan).

### Protein identification

Protein samples were separated by SDS-PAGE, gels were stained with Coomassie Brilliant Blue (CBB) R-250, and two protein bands of interest were excised. The protein bands were then subjected to nano liquid chromatography-tandem mass spectrometry (nano-LC-MS/MS) analysis, and the resulting data were analyzed via a Mascot search against the *B*. *holmesii* database (NCBI taxonomy ID, 35814; available at http://www.ncbi.nlm.nih.gov/Taxonomy/Browser/wwwtax.cgi). The MS analysis was carried out by the Japan Bio Services Co., Ltd. (Saitama, Japan).

### DNA sequencing

The *bipA* gene of *B*. *holmesii* F627 (complement 1618935..1623335, GenBank: AOEW01000001.1) was amplified from *B*. *holmesii* strains and sequenced using appropriate primers (S1 Table). The gene was named as the “outer membrane ligand binding protein” in the GenBank database; however, it was selected as the *bipA* gene based upon sequence similarity to other *Bordetella bipA* genes (see the Results section for more details). Sequencing reactions were carried out with a BigDye Terminator v3.1 Cycle Sequencing Kit (Applied Biosystems, Waltham, MA, USA), and the products were sequenced using a 3130xl Genetic Analyzer (Applied Biosystems) or a 3730 DNA Analyzer (Applied Biosystems).

### Generation of BipA mutant

BipA-deficient mutant was constructed by double-crossover homologous recombination, as described previously but with minor modifications [28]. First, the Δ*bipA* sequence containing a 636-bp deletion was constructed by overlap extension PCR using *B*. *holmesii* BH2 genomic DNA as the template (S1A Fig). Briefly, two DNA fragments, which were 1.4 kbp and 1.2 kbp, were amplified by PCR using the attB1-bipA and MP1-bipA, and MP2-bipA and attB2-bipA primers, respectively (S1 Table). The DNA fragments were joined by overlap extension PCR using the attB1-bipA and attB2-bipA primers, and a 3rd round of PCR was then performed using the attB1-adaptor and attB2-adaptor primers, with the 2nd PCR product being used as the template. The resulting PCR product was cloned into pDONR221 to obtain pDONR221-Δ*bipA* using the adaptor PCR method and the Gateway cloning system (Invitrogen, Waltham, MA, USA). Vectors pDONR221-Δ*bipA* and pABB-CRS2 [29] were then combined using the Gateway cloning system to obtain pABB-Δ*bipA*, which was introduced into *E*. *coli* SM10λ*pir* and trans-conjugated into Sm-resistant *B*. *holmesii* BH2 (BH2Sm^r^). The resulting mutant was designated BH2Sm^r^-ΔBipA. The lack of BipA protein expression in the strain was confirmed by immunoblot analysis (S1B Fig). The BipA mutant had an in *vitro* growth rate that was similar to that of the parental strain BH2Sm^r^.

### Intranasal infection of mice with BipA mutant

To examine the role of BipA during respiratory infection, we employed a mouse intranasal infection model using 4-week-old female BALB/c mice (Japan SLC) [30]. Briefly, strain BH2Sm^r^-ΔBipA was cultured on BG agar containing SM and suspended in casamino acid solution at an OD_650_ of 1.0. The bacterial suspension was diluted 15-fold with casamino acid solution, and 50 μl of the resulting solution (10^7.9^ colony forming unit (CFU)/ml) was instilled intranasally into mice (n = 3 mice per time point) anesthetized by intraperitoneal injection of pentobarbital. After 3, 6, or 24 h, individual lungs of the mice were removed and homogenized in 10 ml of PBS, using an ULTRA-TURRAX tube dispenser and DT-20M sterile tubes (IKA, Staufen, Germany). After sample dilution (10^0^- to 10^4^-fold) in PBS, each homogenate (100 μl) was spread on BG agar containing SM and incubated at 36°C. The number of viable bacteria was calculated using the number of CFU logarithmically transformed. The parental strain BH2Sm^r^ was used as a control, exhibiting a viable cell number of 10^7.6^ CFU/ml. This study was approved by the Animal Research Committee of Kitasato University, and experiments were conducted according to the guidelines of the Ministry of Education, Culture, Sports, Science, and Technology of Japan.

### Quantitative reverse transcriptase-PCR (qRT-PCR)

*B*. *holmesii* strains were cultured on BG agar. Total RNA was isolated using the RNeasy Mini Kit (Qiagen) and treated with RNase-Free DNase (Qiagen) to degrade contaminating DNA. Total RNA (0.1 μg) was reverse-transcribed into cDNA using the PrimeScript RT Master Mix (TaKaRa Bio, Inc.) with random hexamers. The relative levels of *bipA* and *recA* transcripts were determined using SYBR Premix Ex Taq II (TaKaRa Bio) with the ABI PRISM 7500 Fast Real-Time PCR System (Applied Biosystems). The qRT-PCR conditions used were 10 s at 95°C, followed by 40 cycles of 95°C for 3 s and 60°C for 30 s. Primer sets (qbipA-F/qbipA-R and qrecA-F/qrecA-R) were used for *bipA* and *recA* amplification, respectively (S1 Table). Relative *bipA* transcript levels were calculated using the ΔΔCt method and were normalized to those of *recA*, which was used as an internal control for each sample. Data are presented as fold-changes in expression compared to those observed in BH2 bacteria cultured on normal BG agar.

### Statistical analysis

Data are presented as means ± standard deviations. Student’s *t*-test was used for the statistical evaluation of data. *p* < 0.05 was considered statistically significant.

## Results

### Autoagglutination of *B*. *holmesii*

**Fig 1:**
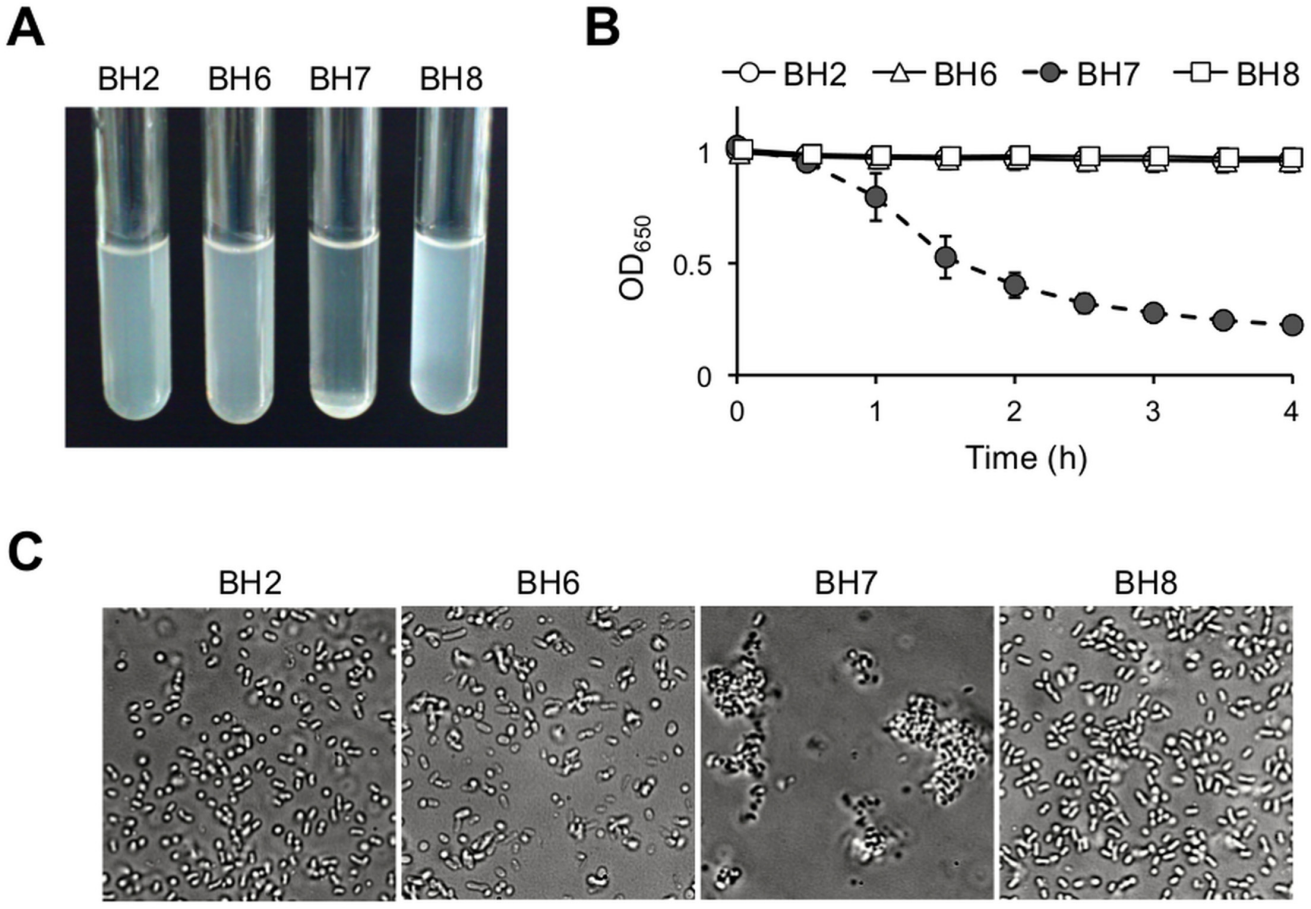
Autoagglutination of *Bordetella holmesii* isolate BH7. (A) The clinical isolates BH2, BH6, BH7, and BH8 were suspended in casamino acid solution and incubated for 4 h at 36°C under static conditions. (B) Time course analysis of the turbidity (OD_650_) of the bacterial suspensions. Data are presented as means ± standard deviations of results obtained from 3 separate experiments performed in triplicate. (C) Microscopic analysis of the morphology of the bacterial suspensions. The suspensions were incubated for 4 h at 36°C on glass slides, and observed by phase-contrast microscopy (objective magnification, 100×).

*B*. *holmesii* isolate BH7 formed aggregates and precipitates in suspension under static conditions (Fig 1A). Indeed, the turbidity of the bacterial suspension significantly decreased from 1.02 to 0.22 (OD_650_) after 4 h of incubation (Fig 1B). In contrast, isolates BH2, BH6, and BH8 did not form aggregates or precipitates (OD_650_ = 0.96–1.01 after 4 h incubation) in suspension. Subsequent microscopic observations confirmed that BH7, but not BH2, BH6, or BH8, formed large aggregates (i.e., bacterial autoagglutination; Fig 1C). Meanwhile, this autoagglutination phenotype was also observed in *B*. *holmesii* strains ATCC 51541 and ATCC 700053, which were isolated from blood samples, but not in isolates BH1, BH3, BH4, BH5, and BH9 (Table 1).

### Biofilm formation by *B*. *holmesii*

**Fig 2:**
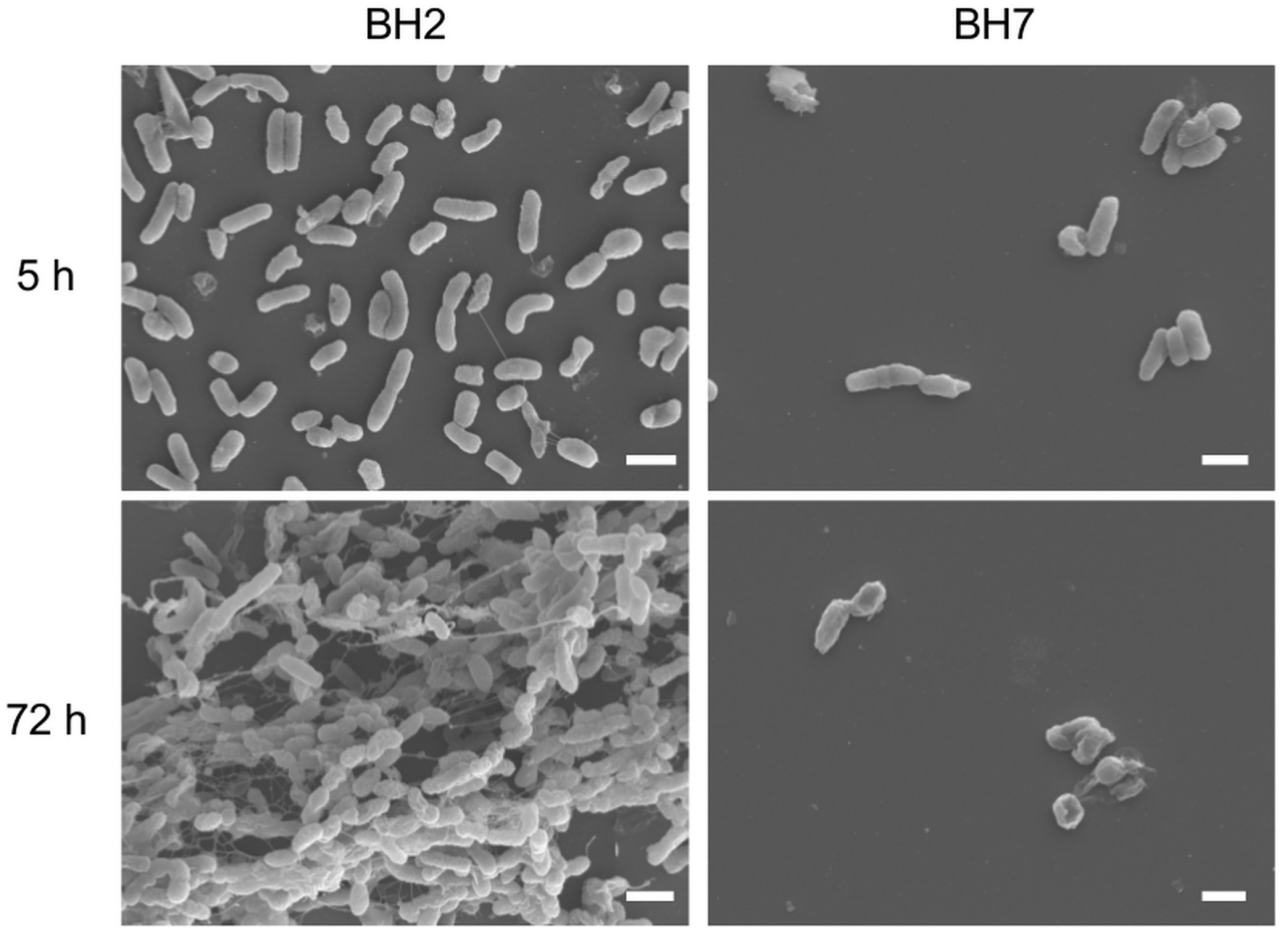
Lack of biofilm formation by *Bordetella holmesii* isolate BH7. The clinical isolates BH2 and BH7 were cultured statically on vertically submerged glass slides in mSS broth. After 5 and 72 h, the cell adherence and the biofilm growth at the air-liquid interface were visualized by SEM. Scale bars, 1 μm

Cell adherence to an abiotic surface and biofilm formation by *B*. *holmesii* isolates were assessed by SEM. The non-autoagglutinating isolate BH2 was observed as scattered cells across the glass slides after culturing for 5 h. At a 72 h culture, the BH2 isolate formed an unusual network of thread-like structures, which radiated from the bacterial surface, indicating that BH2 formed a biofilm *in vitro* (Fig 2). In contrast, the autoagglutinating isolate BH7 adhered to glass slides after culturing for 5 h, but its level of cell adherence was apparently lower than that of BH2. Moreover, the adherent BH7 isolate did not form biofilms on glass slides during the 72 h cultivation. These results suggested that BH7 failed to form biofilms, due to its low adherence to the abiotic surface.

### Identification of BipA associated with bacterial autoagglutination and a defect in biofilm formation

**Fig 3:**
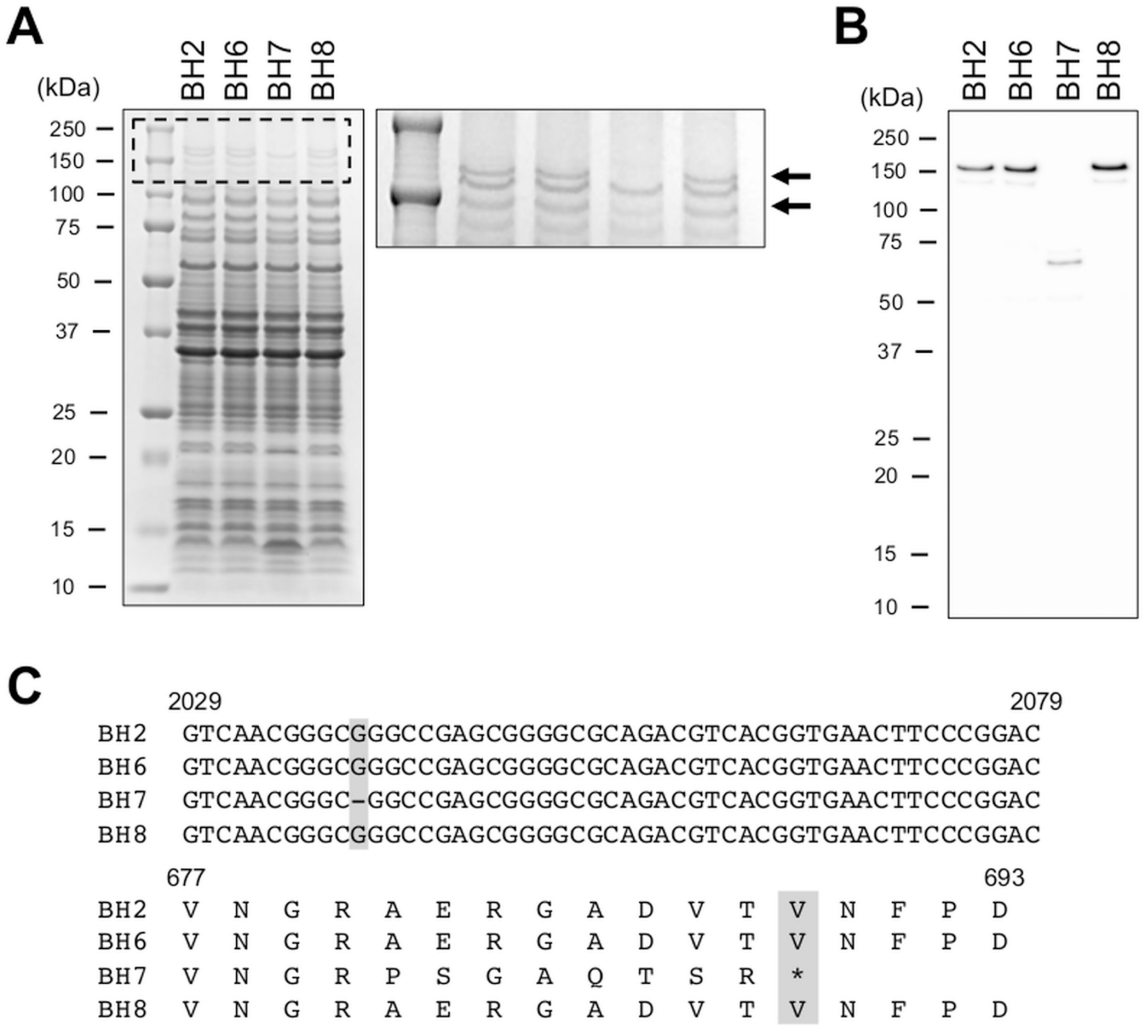
Lack of BipA production in *Bordetella holmesii* isolate BH7. (A) Comparison of the protein expression profiles of the clinical isolates BH2, BH6, BH7, and BH8. Total protein (20 μg) was subjected to SDS-PAGE followed by CBB staining (left panel). The dashed-boxed area is enlarged (right panel). The arrows indicate the bands that were identified as BipA by nano-LC-MS/MS analysis. (B) Analysis of BipA production in *B*. *holmesii* isolates. Total protein (2 μg) was subjected to immunoblot analysis with anti-BipA antisera. (C) Comparison of BipA sequences among the isolates. The *bipA* gene of BH7 encodes a deletion of a guanine (G) at nucleotide position 2039 (gray box). The deletion mutation generates a premature stop codon at nucleotide position 2066. The translation stop site (amino acid 689) is shown in the deduced amino acid sequence (BH7, gray box).

To identify the protein(s) associated with bacterial autoagglutination, the total protein expression profile of *B*. *holmesii* BH7 was analyzed by SDS-PAGE and compared with that of BH2, BH6, and BH8 (non-autoagglutinating isolates). There were 2 protein bands (approximately 150-kDa) that were present in the BH2, BH6, and BH8 samples but absent from the BH7 lysate (Fig 3A). Subsequent nano-LC-MS/MS and Mascot search analyses of the bands present in the BH2 sample identified the two proteins as an outer membrane ligand binding protein (GenBank: EMD74886.1) of *B*. *holmesii* F627. Furthermore, the amino acid sequence of the protein was 57−59% identical to that of BipA of *B*. *bronchiseptica* and *B*. *pertussis*, and the protein contained a conserved intimin domain and seven 90-amino acid repeats similar to those of other *Bordetella* BipA proteins [21]. We therefore designated the outer membrane ligand binding protein as BipA.

As shown in Fig 3B, immunoblot analysis using anti-BipA antisera detected two protein bands (corresponding to BipA) in isolates BH2, BH6, and BH8, but not BH7. Instead, a protein band that was approximately 70-kDa was detected in BH7. Sequencing analysis revealed that the *bipA* gene of isolate BH7 contained a guanine (G) deletion at nucleotide position 2039 (c.2039delG), resulting in the generation of a premature stop codon at nucleotide position 2066 (amino acid 689, p.Ala681ProfsX9) (Fig 3C). As such, our immunoblot analysis might have detected the truncated form BipA in isolate BH7.

### Autoagglutination of constructed BipA mutant

**Fig 4:**
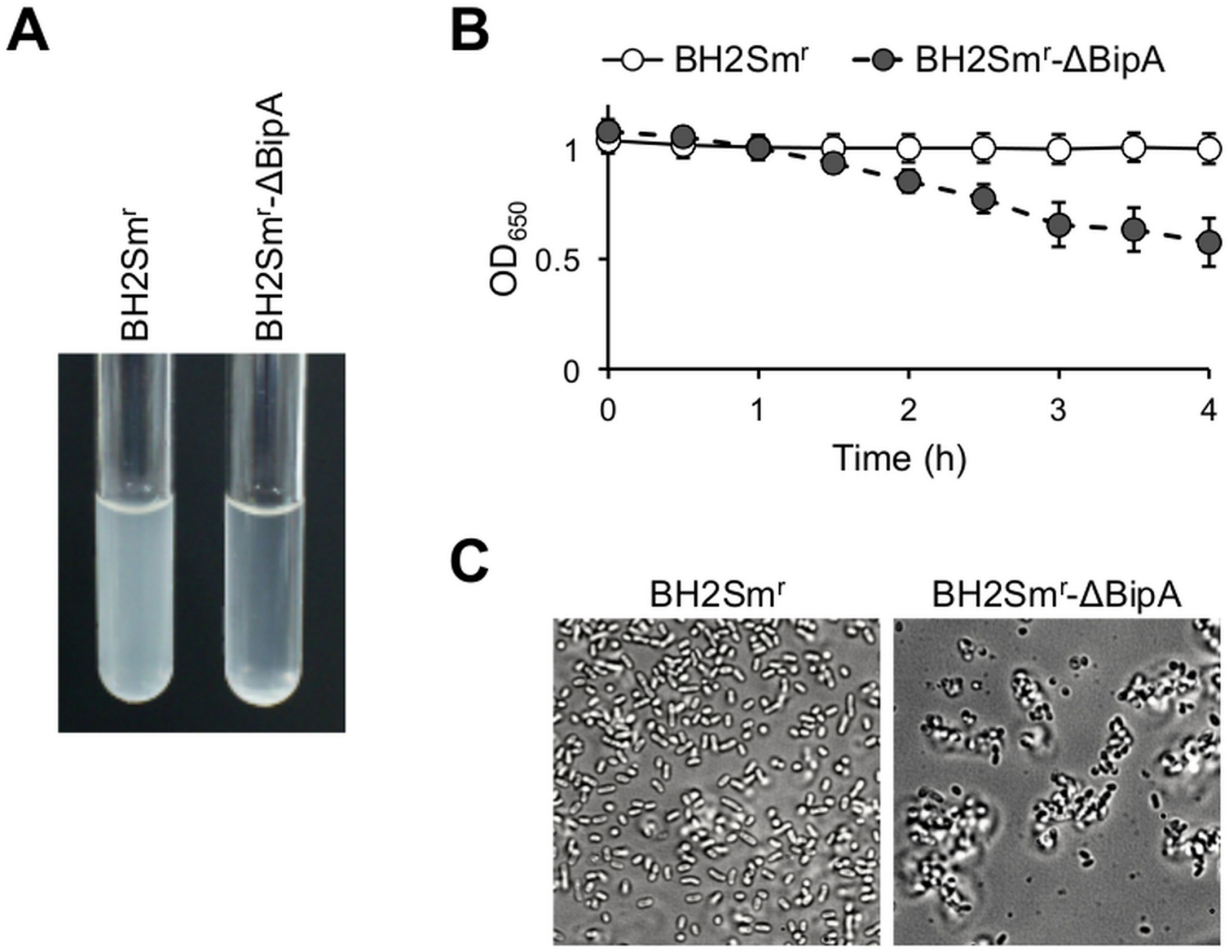
Autoagglutination of BipA mutant. (A) BipA mutant BH2Sm^r^-ΔBipA and its parental strain, BH2Sm^r^, were suspended in casamino acid solution and incubated for 4 h at 36°C under static conditions. (B) Time course analysis of the turbidity (OD_650_) of the bacterial suspensions. Data are presented as the means ± standard deviations of results obtained from 3 separate experiments performed in triplicate. (C) Microscopic analysis of the morphology of the bacterial suspensions. The suspensions were incubated for 4 h at 36°C on glass slides, and observed by phase-contrast microscopy (objective magnification, 100×).

As shown in Fig 4A, the BipA-mutant strain BH2Sm^r^-ΔBipA, constructed by homologous recombination, formed aggregates and precipitates in suspension under static conditions. Specifically, the turbidity of the bacterial suspension significantly decreased from 1.08 to 0.58 (OD_650_) after a 4 h of incubation. Meanwhile, the parental strain, BH2Sm^r^, did not form aggregates or precipitates during the 4 h incubation period (OD_650_ = 1.00 ± 0.07; Fig 4B). Microscopic observations subsequently detected high levels of autoagglutination by the BH2Sm^r^-ΔBipA cells (Fig 4C). Notably, the bacterial aggregates were easily broken into single cells by repeat pipetting.

### Biofilm formation by constructed BipA mutant

**Fig 5:**
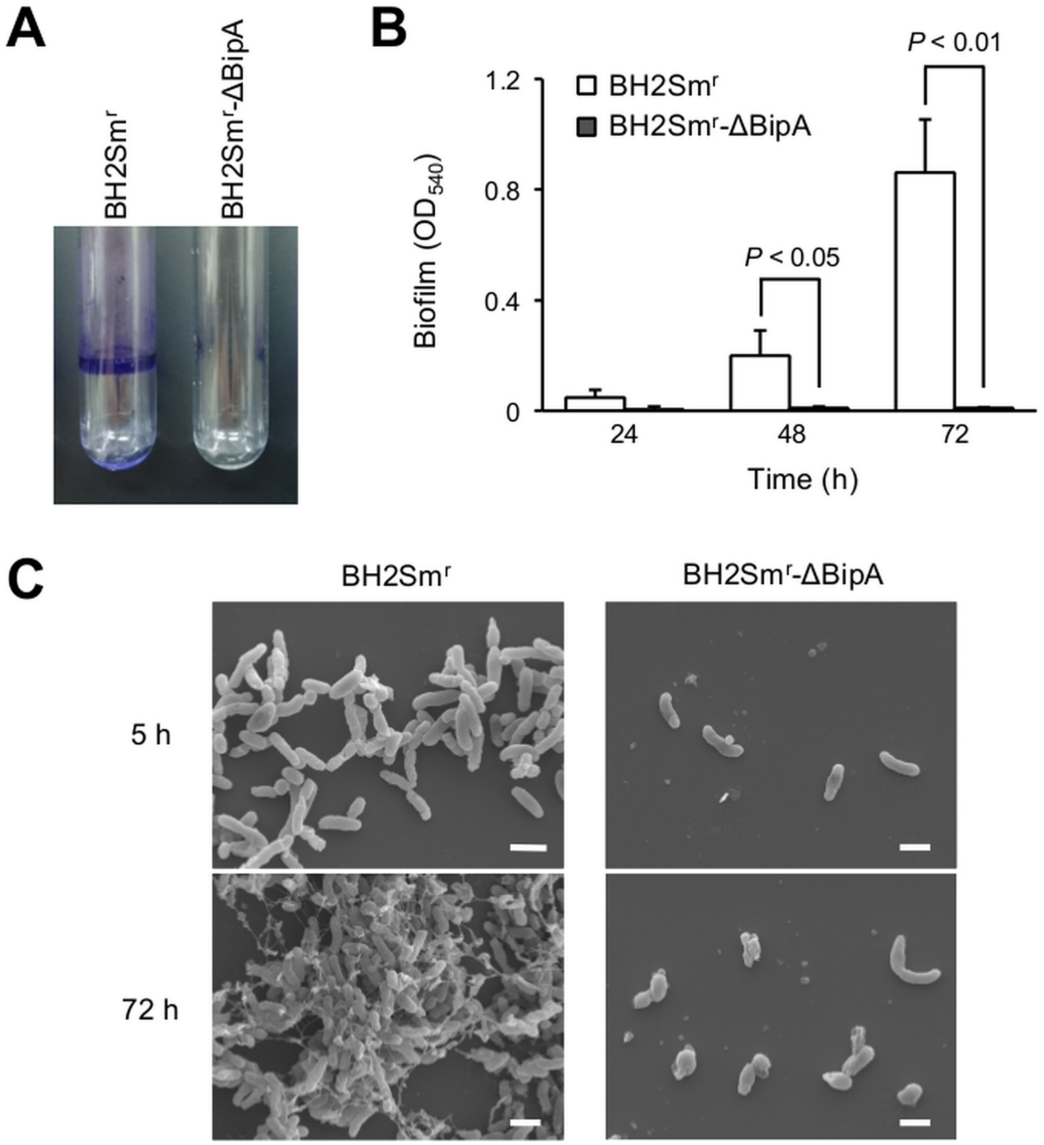
Lack of biofilm formation by BipA mutant. (A) BipA mutant strain BH2Sm^r^-ΔBipA and its parental strain, BH2Sm^r^, were cultured in mSS broth for 72 h under static conditions, and biofilm-forming bacteria were stained with crystal violet. (B) Time course analysis of the levels of biofilm biomass production. The biofilm-associated crystal violet was solubilized and quantified after 24, 48, and 72 h incubation. Data are presented as means ± standard deviations of the results obtained from 3 separate experiments performed in triplicate. Statistical significance was determined using Student’s *t*-test. (C) SEM analysis of biofilm formation. BH2Sm^r^-ΔBipA and BH2Sm^r^ were cultured statically on vertically submerged glass slides in mSS broth. After 5 and 72 h, cell adherence and biofilm growth at the air-liquid interface were visualized by SEM. Scale bars, 1 μm

We cultured BH2Sm^r^-ΔBipA in polystyrene tubes under static conditions and examined the levels of cell adherence to the polystyrene surface. As shown in Fig 5A, BH2Sm^r^-ΔBipA was defective in its ability to form biofilms. Indeed, the biofilm biomass of this strain remained near the background level of staining after culturing for 72 h (Fig 5B). In contrast, the biofilm biomass of BH2Sm^r^ significantly increased over time, reaching 0.86 (OD_540_) after 72 h of cultivation. Subsequent SEM observations confirmed that BH2Sm^r^ formed biofilms after culturing for 72 h, while BH2Sm^r^-ΔBipA adhered poorly to glass slides and failed to form biofilms during the 72 h cultivation period (Fig 5C). These data suggested that the autoagglutination phenotype of BH2Sm^r^-ΔBipA inhibited the strain’s ability to adhere to the abiotic surface and, consequently failed to form biofilms *in vitro*.

### Initial colonization of constructed BipA mutant in murine lungs

**Fig 6:**
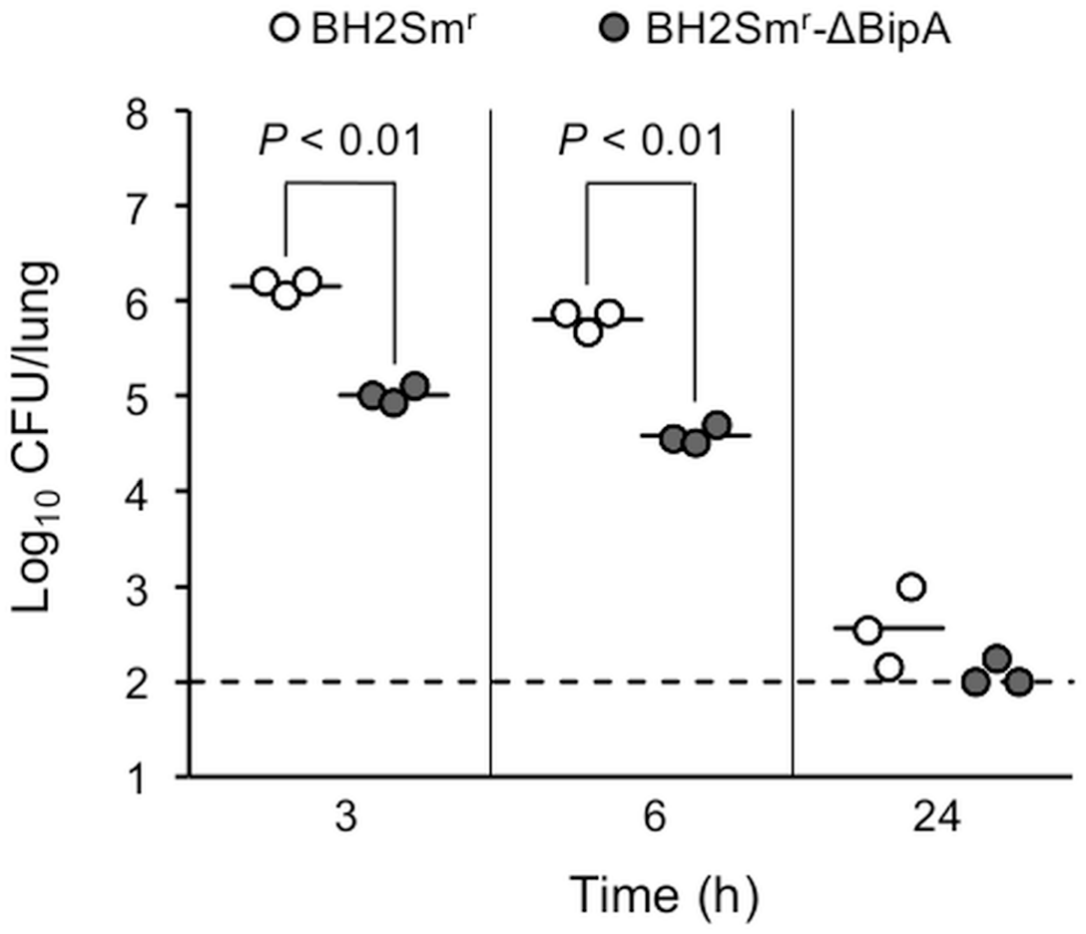
Initial colonization of BipA mutant in murine lungs. Groups of three BALB/c mice were intranasally infected with the BipA mutant BH2Sm^r^-ΔBipA or its parental strain BH2Sm^r^. The colonization levels were assessed after 3, 6, and 24 h. Each symbol represents one mouse, and horizontal lines represent the mean number of CFU recovered. Statistical significance was determined using Student’s *t*-test. The dashed line indicates the lower limit of detection (100 CFU/lung). The lung CFUs in two mice infected with BH2Sm^r^-ΔBipA were below the limit of detection at 24 h post-infection.

Mice were infected with BH2Sm^r^-ΔBipA or BH2Sm^r^, and after 3, 6, and 24 h the numbers of viable bacteria recovered from the lungs of the infected animals (n = 3) were compared. As shown in Fig 6, the average levels of colonization by BH2Sm^r^-ΔBipA were significantly lower than those of BH2Sm^r^ (log_10_ CFU, *P* < 0.01) at 3 and 6 h post-infection: 10^5.0^ CFU versus 10^6.2^ CFU at 3 h, and 10^4.6^ CFU versus 10^5.8^ CFU at 6 h, respectively. In contrast, both lung CFUs of BH2Sm^r^-ΔBipA and BH2Sm^r^ drastically decreased at 24 h post-infection, and the number of CFUs was near or below the detection limit of 100 CFU (not applicable to the Student’s *t*-test). The average recovery rates of BH2Sm^r^-ΔBipA were 2.8% and 1.1% at 3 and 6 h post-infection, respectively, while those of BH2Sm^r^ were 77% and 35%, respectively. Significant differences were observed in the initial colonization levels at 3 and 6 h post-infection between the BipA mutant and its parental strain.

### Relationship between BipA production and biofilm formation in other *B*. *holmesii* strains

**Fig 7:**
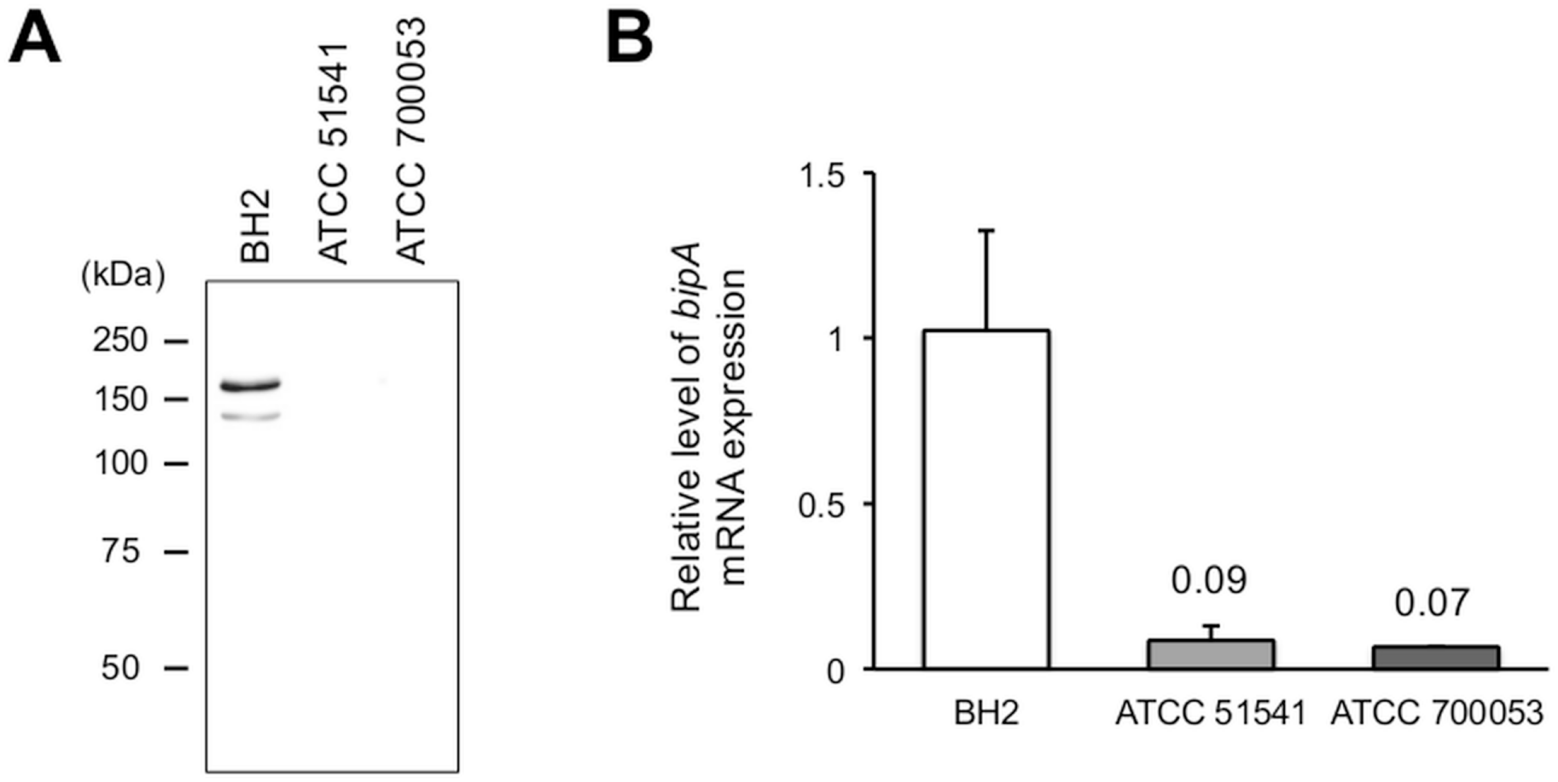
Lack of BipA protein and transcript expression in *Bordetella holmesii* ATCC strains. (A) *B*. *holmesii* BH2, ATCC51541, and ATCC700053 were cultured for 4 days on BG agar. Total protein (2 μg) was subjected to immunoblot analysis with anti-BipA antisera. (B) After culturing for 3 days on BG agar, total RNA was isolated, reverse transcribed into cDNA, and then analyzed by qRT-PCR analysis. Relative *bipA* transcript levels were calculated using the ΔΔCt method and were normalized to those of *recA*. The recA transcript was used as an internal control for each sample. Data are presented as fold-changes in expression compared to those observed in BH2. The means ± standard deviations of results obtained from 3 separate experiments are shown.

We examined the levels of BipA production in other *B*. *holmesii* strains by immunoblot analysis. Interestingly, BipA production was detected in each of 5 isolates (BH1, BH3, BH4, BH5, and BH9); however, the protein was not detected in strains ATCC 51541 or ATCC 700053, which were isolated from blood infections. Likewise, the two ATCC strains were the only isolates that failed to exhibit *in vitro* biofilm formation. While sequence analyses revealed that these strains encoded an intact *bipA* gene, qRT-PCR detected markedly lower levels of *bipA* gene expression (0.07- to 0.09-fold) in the ATCC strains than in BH2 (Fig 7). Interestingly, sequence analysis identified some mutations in *bipA* upstream region and *bvgA* gene of the ATCC 700053 strain (S2 Fig). Although the ATCC 51541 strain also was found to have an insertion mutation in the *bvgA* gene [31], the relation between these mutations and BipA downregulation is presently unknown. Taken together, our findings indicate that BipA production is downregulated at the transcriptional level in ATCC 51541 and ATCC 700053. The characteristics of clinical and ATCC strains are summarized in Table 1.

## Discussion

In the present study, we analyzed a *B*. *holmesii* isolate exhibiting autoagglutination and found that this phenotype was the result of a defect in BipA production. Indeed, a BipA-deficient mutant generated by homologous recombination also exhibited the autoagglutination phenotype. Notably, the BipA mutant, as well as other autoagglutinating *B*. *holmesii* isolates, did not show biofilm formation. Our findings therefore suggest that BipA production plays essential roles in preventing bacterial autoagglutination and indirectly promoting biofilm formation in *B*. *holmesii*. To the best of our knowledge, this is the first report to demonstrate a function for the *B*. *holmesii* BipA protein.

Biofilms are microbial communities in which bacteria adhere to each other and to biotic or abiotic surfaces. Biofilm development occurs through multiple sequential processes, including adherence to a surface, microcolony formation, and establishment of a 3-dimensional structure. Three classical *Bordetella* species, *B*. *pertussis*, *B*. *parapertussis*, and *B*. *bronchiseptica*, are capable of forming biofilms on abiotic surfaces and within the mouse respiratory tract [25, 27, 32–34]; however, the biofilm-forming capacity of *B*. *holmesii* was previously unreported. The present study clearly demonstrates that BipA-producing *B*. *holmesii* isolates formed biofilms *in vitro*. Conversely, strains that were deficient in BipA production adhered poorly to an abiotic surface consequently failed to form biofilms, likely due to the high levels of bacterial autoagglutination associated with these strains. These findings therefore suggest that BipA may act as an autoagglutination inhibitor and contributes indirectly to biofilm formation in *B*. *holmesii*. In contrast, a previous study showed that while *B*. *bronchiseptica* exhibited autoagglutination under Bvg^i^ phase, this phenotype was not BipA-dependent [21], indicating that there are functional differences between the BipA proteins of *B*. *bronchiseptica* and *B*. *holmesii*. BipA localizes to the outer membrane of the bacterial cell, and the C-terminus of the protein is exposed to the environment [21]. The C-terminal domain (278 amino acids) of *B*. *bronchiseptica* BipA is nearly identical to those of BipA proteins produced by *B*. *pertussis* and *B*. *parapertussis* (>88% identity). Conversely, the C-terminal domain (294 amino acids) of *B*. *holmesii* BipA exhibits much lower sequence identity (42–43%) to those of the classical *Bordetella* species. Thus, this distinct C-terminal domain may play a role in the anti-autoagglutination effect of BipA in *B*. *holmesii*, perhaps by inhibiting cell-to-cell binding. While this is a potential explanation for *B*. *holmesii* autoagglutination, we have not excluded other possible explanations. For instance, the absence of BipA may allow the expression of an unknown factor that causes autoagglutination, and the absence of BipA may affect the surface charge or hydrophobicity, leading to the autoagglutination. Further studies are required to elucidate the molecular mechanism governing *B*. *holmesii* autoagglutination.

In our autoagglutination assay, the constructed BipA mutant BH2Sm^r^-ΔBipA showed slower autoagglutination kinetics than that of the naturally occurring BipA-deficient strain BH7 (Figs 1 and 4). Our immunoblot analysis revealed that BH2Sm^r^-ΔBipA does not produce any detectable truncated BipA, which allows for a partially functional BipA (S1B Fig). These findings suggest that the strength of autoagglutination may depend on the strain used. In addition, we observed that the BipA^+^ back-mutant (BH7Sm^r^-BipA^+^) showed slower autoagglutination kinetics than that of its parental strain BH7Sm^r^ (S3 Fig). However, the restoration of agglutinBipA production failed to completely abrogate the autoagglutination. This observation may have been due to the acquisition of Sm resistance, as the autoagglutination kinetics of BH7Sm^r^ was faster than that of the parental isolate BH7. These results support our hypothesis that BipA is associated with preventing autoagglutination in *B*. *holmesii*.

In a previous study, a BipA homolog was identified and designated as Bordetella colonization factor A (BcfA), which contains an intimin domain (similar to the BipA protein) in its N-terminal region, in *B*. *bronchiseptica* [22]. In this study, we identified an outer membrane ligand binding protein that was absent from an autoagglutinating isolate of *B*. *holmesii* (Fig 3A). The amino acid sequence of the protein was 57% and 44% identical to that of *B*. *bronchiseptica* BipA (GenBank: CAE32799.1) and BcfA (GenBank: CAE30611.1), respectively. In addition, the outer membrane ligand binding protein contained not only an intimin domain but also a highly conserved 90-amino acid repeats, similar to BipA of *B*. *bronchiseptica* and *B*. *pertussis* [21]. We therefore designated the *B*. *holmesii* protein as BipA, but not BcfA.

In the present study, the BipA mutant BH2Sm^r^-ΔBipA was associated with lower levels of initial colonization in murine lungs than the parental strain BH2Sm^r^, suggesting that BipA may function as an adhesion in *B*. *holmesii*. However, we also observed that the BipA mutant adhered to cultured human lung cells as large aggregates via autoagglutination (S4 Fig). Thus, while there is no evidence that the *B*. *holmesii* BipA acts as an adhesin, expression of the protein appears to be crucial during early stages of lung colonization. Consistent with this conclusion, previous studies demonstrated that the *B*. *bronchiseptica* BipA was not directly involved in respiratory tract colonization [21]. Furthermore, Vergara-Irigaray et al. suggested that BipA is not strictly required for respiratory tract colonization by *B*. *pertussis*, but is instead necessary immediately before and/or after a transmission event [35]. In regard to our observations, one possible explanation for the lower lung colonization level of the BipA mutant is that bacterial aggregates may be less efficient at adhering to the respiratory epithelium than single cells. Consequently, such aggregates would be easily cleared from respiratory tract sites. Alternatively, bacterial aggregates may be more rapidly eliminated from respiratory tract sites or efficiently killed by phagocytes than individual cells [36]. BipA may have the ability to promote suppression or evasion of the host innate immune system.

In *B*. *pertussis*, *B*. *parapertussis*, and *B*. *bronchiseptica*, BipA is characterized as a Bvg-intermediate phase protein. These BipA proteins are maximally expressed under intermediate Bvg^i^ phase, but are expressed at very low or undetectable levels during the virulent Bvg^+^ and avirulent Bvg^-^ phases, respectively [21, 37]. Bvg phase transitions are dependent on environmental signals such as culture temperature and chemical modulators (MgSO_4_ and nicotinic acid). In our preliminary study, however, we found that the transcript and protein levels of *B*. *holmesii* BipA were largely unaffected by the presence or absence of chemical modulators (S5 Fig). While *B*. *holmesii* encodes a BvgAS sensory transduction system [18, 30], it is possible that, unlike in other *Bordetella* species, BipA may not be regulated by the BvgAS system in *B*. *holmesii*. In addition, we confirmed that putative Bvg binding sites are not present in the upstream region of *bipA*.

*B*. *holmesii* causes both invasive and respiratory diseases in humans, and the number of cases of respiratory infection has increased in the last decade worldwide [6, 8–13]. Genome analyses revealed that circulating *B*. *holmesii* strains are closely related, and that important virulence genes that are present in *B*. *pertussis* are not found in *B*. *holmesii* [16, 17]. Moreover, no traits specific to *B*. *holmesii* respiratory or blood isolates were identified previously [38]. Notably, while each of the respiratory isolates tested in the present study produced BipA, 3 of the 5 invasive isolates tested, including 2 ATCC strains, did not. These findings suggest that BipA may play an important role during *B*. *holmesii* respiratory infections, but may be dispensable during invasive infections. This hypothesis is supported by our observation that the BipA-deficient mutant exhibited a lower initial colonization level than did its parental strain (Fig 6). However, because five of the 6 *B*. *holmesii* respiratory isolates tested were collected from the same outbreak in Japan [9], further study, using a larger number of *B*. *holmesii* isolates, is required to fully test this hypothesis.

Recently, BipA was identified as one of the most abundant proteins in *B*. *pertussis* biofilms [39]. Furthermore, immunization with recombinant *B*. *pertussis* BipA protected against *B*. *pertussis* infection in an animal model, suggesting that BipA may be potentially considered for inclusion as an antigen in pertussis vaccines to improve their effectiveness. Meanwhile, current pertussis vaccines confer little protection against *B*. *holmesii* infection [14]. As such, the development of a novel vaccine that is effective against *B*. *holmesii* will be necessary. Given that BipA was produced by all *B*. *holmesii* respiratory isolates tested in this study, this protein may also comprise an ideal antigen for use in the production of a vaccine to prevent *B*. *holmesii* respiratory infections. Indeed, attempts to develop a *B*. *holmesii* vaccine using BipA are now in progress.

In conclusion, BipA plays an essential role in preventing autoagglutination and indirectly promoting biofilm formation in *B*. *holmesii*. Bacterial biofilms are important contributors to chronic and persistent diseases. Because *B*. *holmesii* BipA production was detected in all respiratory isolates tested, we consider BipA a crucial virulence factor during respiratory infections by this organism. However, further studies using additional isolates are needed to elucidate the role of BipA in the pathogenesis of *B*. *holmesii* infections.

## Supporting Information

S1 Fig Construction and characterization of *Bordetella holmesii* BipA mutant. (A) Physical map of the *bipA* gene of the BipA-deficient strain BH2Sm^r^-ΔBipA, which was derived from BH2Sm^r^. (B) Total protein (2 μg) was extracted from strains BH2, BH2Sm^r^, and BH2Sm^r^-ΔBipA and subjected to immunoblot analysis using anti-BipA antisera.
(EPS)

S2 Fig Comparison of sequences among *Bordetella holmesii* isolates and ATCC strains. (A) Two guanines are substituted for adenine at nucleotide positions -242 and -231 in the *bipA* upstream region of the ATCC700053 strain (gray box). (B) The *bvgA* gene of the ATCC51541 strain encodes an adenine insertion at nucleotide position 212 (gray box), resulting in a premature stop codon at nucleotide position 279. A thymine is substituted for cytosine at nucleotide position 155 in the *bipA* gene of the ATCC700053 strain (gray box).
(EPS)

S3 Fig Construction and autoagglutination of BipA^+^ back-mutant. The BipA^+^ back-mutant BH7Sm^r^-BipA^+^ was constructed from BipA-deficient isolate BH7 by double-crossover homologous recombination. A 2.5-kbp DNA fragment encoding the intact *bipA* gene was amplified by PCR with the attB1-BH2-bipA and attB2-BH2-bipA primers (S1 Table) using BH2 genomic DNA as the template. The resulting PCR product was cloned into pDONR221 and then combined with pABB-CRS2 using the Gateway cloning system to obtain pABB-*bipA*, which was trans-conjugated into BH7Sm^r^ via *E*. *coli* SM10λ*pir*. The resulting mutant was designated BH7Sm^r^-BipA^+^. (A) BipA production in the strain was confirmed by immunoblot analysis using anti-BipA antisera. (B) BH7Sm^r^-BipA^+^, its parental strain BH7Sm^r^, and BH7 were suspended in casamino acid solution and incubated for 4 h at 36°C under static conditions. The turbidity (OD_650_) of the bacterial suspensions was measured every 30 min.
(EPS)

S4 Fig Adherence of BipA mutant to A549 cells. A549 cells were grown in DMEM containing 1% FBS on a glass coverslips in 6-well tissue culture plates. After a 24 h culture, the medium was removed and replaced with fresh DMEM. BH2, BH2Sm^r^, or BH2Sm^r^-ΔBipA (6 × 10^7^ CFU in 1% casamino acid solution) was added to the A549 cells at a multiplicity of infection (MOI) of 200. The plates were centrifuged at 200 × *g* for 5 min, then incubated at 36°C. After 2 h, the cells were washed, fixed with 4% paraformaldehyde, permeabilized with 0.2% TritonX-100, and quenched with 50 mM ammonium chloride. The fixed cells were incubated with a *B*. *holmesii* BH7-specific primary antibody followed by a fluorescein isothiocyanate (FITC)-conjugated secondary antibody (green). Actin and nuclear DNA were visualized by rhodamine phalloidin (red) and TO-PRO3 (blue) staining, respectively. Subsequently, the bacterial cells were examined by fluorescence microscopy using an Olympus IX83 (objective magnification, 100×).
(EPS)

S5 Fig Effect of MgSO_4_ and nicotinic acid on BipA protein and transcript expression. (A) *Bordetella holmesii* BH2 was cultured for 4 days on BG agar supplemented with MgSO_4_ (10 or 50 mM) or nicotinic acid (0.5 or 20 mM). Total protein (2 μg) was extracted from the bacterial cells and analyzed by immunoblot using anti-BipA antisera. (B) After culturing for 3 days on BG agar supplemented with MgSO_4_ (10 or 50 mM) or nicotinic acid (0.5 or 20 mM), the relative *bipA* transcriptional level was examined according to the method described in S2 Fig. Data are presented as fold-changes in expression compared to those observed in BH2 bacteria cultured on normal BG agar, and the means ± standard deviations of results obtained from 3 separate experiments are shown.
(EPS)

S1 Table Primers used in this study. (DOCX)
