# Supplementary material for: BipA Is Associated with Preventing Autoagglutination and Promoting Biofilm Formation in Bordetella holmesii
Source: PLoS One. 2016 Jul 22;11(7):e0159999. doi: 10.1371/journal.pone.0159999 (PMC4957798; doi:10.1371/journal.pone.0159999)
Supplement: S1 Table — (DOCX) [file pone.0159999.s006.docx]

| **S1 Table.** Primers used in this study | | |
| --- | --- | --- |
| Designation | Primer name | Sequence (5´ to 3´) |
| DNA sequencing | bipA-F | TGCAAGCGTGCCAGAAAACG |
|  | bipA-R | TGTCGAGAACAGACCTAGCCA |
|  | bipA-F2 | CAATCCCTTGCACAGGCT |
|  | bipA-F3 | CATGTGGACTATTACGAC |
|  | bipA-F4 | GTGCGTTCGCTGGAAGTC |
|  | bipA-F5 | GTGGTCGCTGCCGATGAC |
|  | bipA-F6 | CGATGATGCTCCGGATAC |
|  | bipA-F7 | GACCGTCAAAGGTCAGAC |
|  | bipA-F8 | GACCGATGGAAGCTTCAC |
|  | bipA-R2 | AGCCTGTGCAAGGGATTG |
|  | bipA-R3 | GTCGTAATAGTCCACATG |
|  | bipA-R4 | GACTTCCAGCGAACGCAC |
|  | bipA-R5 | GTCATCGGCAGCGACCAC |
|  | bipA-R6 | GTATCCGGAGCATCATCG |
|  | bipA-R7 | GTCTGACCTTTGACGGTC |
|  | bipA-R8 | GTGAAGCTTCCATCGGTC |
| Generation of BipA mutant | attB1-bipA | AAAAAGCAGGCTGCATAACCAGAACAGCCGCC |
|  | MP1-bipA | CTGAACATGCCATCGTCGCCCTACTAGTCGGTGGTGATAAGGCGGA |
|  | MP2-bipA | TCCGCCTTATCACCACCGACTAGTAGGGCGACGATGGCATGTTCAG |
|  | attB2-bipA | AGAAAGCTGGGTTCTTGGTCGGTTGGCTCGTG |
|  | attB1-adaptor | GGGGACAAGTTTGTACAAAAAAGCAGGCT |
|  | attB2-adaptor | GGGGACCACTTTGTACAAGAAAGCTGGGT |
| Generation  of BipA^+^  back-mutant | attB1-BH2-bipA | AAAAAGCAGGCTTCCAATACAGTCCTGCCTTC |
|  | attB2-BH2-bipA | AGAAAGCTGGGTGCGCCGTTTTATCTACCTTG |
| Generation of rBipA | bipA R1-F | ATCATCATCATCATATGATTCCCACGCTGGGCCCA |
|  | bipA R1-R | GCTTGAATTCGGATCCTCAGGTTTTCGCCTCGGTGAC |
|  | bipA R3-F | ATCATCATCATCATATGACGGTAAGTGGCAAGGCA |
|  | bipA R3-R | GCTTGAATTCGGATCCTCAGTTGTGCGGCGGCTC |
| qRT-PCR | qbipA-F | TACATCGAGGTGACTTCCCAAG |
|  | qbipA-R | GCAGGCGCCGTTTTATCTAC |
|  | qrecA-F | CAAACTGACCGCCACTATCAAG |
|  | qrecA-R | AGGCGCACAGAAGCATAGAAC |
